# Functional Characterisation of Germinant Receptors in *Clostridium botulinum* and *Clostridium sporogenes* Presents Novel Insights into Spore Germination Systems

**DOI:** 10.1371/journal.ppat.1004382

**Published:** 2014-09-11

**Authors:** Jason Brunt, June Plowman, Duncan J. H. Gaskin, Manoa Itchner, Andrew T. Carter, Michael W. Peck

**Affiliations:** Gut Health and Food Safety, Institute of Food Research (IFR), Norwich Research Park, Colney, Norwich, Norfolk, United Kingdom; Tufts University, United States of America

## Abstract

*Clostridium botulinum* is a dangerous pathogen that forms the highly potent botulinum toxin, which when ingested causes a deadly neuroparalytic disease. The closely related *Clostridium sporogenes* is occasionally pathogenic, frequently associated with food spoilage and regarded as the non-toxigenic equivalent of Group I *C. botulinum*. Both species form highly resistant spores that are ubiquitous in the environment and which, under favourable growth conditions germinate to produce vegetative cells. To improve the control of botulinum neurotoxin-forming clostridia, it is imperative to comprehend the mechanisms by which spores germinate. Germination is initiated following the recognition of small molecules (germinants) by a specific germinant receptor (GR) located in the spore inner membrane. The present study precisely defines clostridial GRs, germinants and co-germinants. Group I *C. botulinum* ATCC3502 contains two tricistronic and one pentacistronic GR operons, while *C. sporogenes* ATCC15579 has three tricistronic and one tetracistronic GR operons. Insertional knockout mutants, allied with characterisation of recombinant GRs shows for the first time that amino acid stimulated germination in *C. botulinum* requires two tri-cistronic encoded GRs which act in synergy and cannot function individually. Spore germination in *C. sporogenes* requires one tri-cistronic GR. Two other GRs form part of a complex involved in controlling the rate of amino-acid stimulated germination. The suitability of using *C. sporogenes* as a substitute for *C. botulinum* in germination studies and food challenge tests is discussed.

## Author Summary

*Clostridium botulinum* is a dangerous pathogen that forms the deadly botulinum neurotoxin. Strains of *C. botulinum* are present in the environment as spores. Under suitable conditions, the dormancy of the bacterial spore is broken, and germination occurs. Germination is initiated following the recognition of small molecules by a specific germinant receptor (GR) located within spores. Currently, the identification and characterisation of these GRs remains unknown, but is critical if strategies are to be developed to either prevent spore germination altogether, or to germinate all the spores and then inactivate the emergent sensitive vegetative cells. The present study has characterised two functionally active GRs in *C. botulinum* which act in synergy and cannot function individually, and a related functionally active GR in *C. sporogenes*. These GRs respond to amino acids. Other GRs appear to form part of a complex involved in controlling the speed of germination, or are not functionally active. This study provides new insights into the mechanisms involved in germination and will allow us to develop new strategies to control this deadly pathogen.

## Introduction

*Clostridium botulinum* and *Clostridium sporogenes* are closely related anaerobic spore-forming bacteria. *C. botulinum* is a dangerous pathogen that forms the deadly botulinum neurotoxin. This is the most potent toxin known, as little as 30–100 ng can be fatal [1]. Eight distinct types of botulinum neurotoxin (types A to H), and more than thirty different neurotoxin sub-types (e.g. sub-types A1 to A5) are recognised [2]–[5]. The botulinum neurotoxins are 150 kD proteins with zinc-endopeptidase activity that block acetylcholine transmission in cholinergic nerves, leading to a floppy paralysis known as botulism, that may prove fatal to both humans and animals [6], [7]. The most frequently reported types of human botulism are foodborne botulism, infant botulism and wound botulism [1], [8]. Foodborne botulism is an intoxication caused by consumption of neurotoxin formed by *C. botulinum* following spore germination and growth of vegetative cells in food. Infant and wound botulism are infections involving spore germination, growth of vegetative cells and neurotoxin formation in the gut of young infants and in deep wounds (often associated with drug abuse), respectively. Botulinum neurotoxins are also important pharmaceuticals used to treat a range of localised conditions e.g. blepharospasm, hemifacial spasm, and for cosmetic purposes [9].

*C. botulinum* is a heterogeneous species that comprises a complex of four distinct groups of bacteria that share the common property of forming the botulinum neurotoxin [3], [10], [11]. Group I (proteolytic) *C. botulinum* is associated with foodborne botulism, infant botulism and wound botulism, and forms one or more neurotoxins of types A, B, F or H [3], [5], [10], [11]. Strains of Group I *C. botulinum* that form type A1 neurotoxin have received the most attention to date because they are often associated with botulism in humans, the extreme potency of the type A1 neurotoxin, and due to the use of type A1 neurotoxin as a pharmaceutical [1], [12]–[15]. Indeed, the first *C. botulinum* genome to be sequenced was that of Group I *C. botulinum* type A1 strain ATCC3502 [16].

*C. sporogenes* is occasionally pathogenic [17], a significant cause of food spoilage [18], and because of its strong physiological similarity to Group I *C. botulinum* is very widely used as a surrogate for this organism in demonstrating the effectiveness of food preservation processes [19], [20]. Genome sequencing, whole genome analysis using DNA microarrays, and other typing methods have confirmed the close genetic relationship of *C. sporogenes* and Group I *C. botulinum* [2], [21]–[23]. The formation of botulinum neurotoxin is used to distinguish strains of Group I *C. botulinum* from those of *C. sporogenes* [19].

Strains of Group I *C. botulinum* and *C. sporogenes* are both present in the environment as spores. This highly resistant dormant state enables survival in adverse conditions (e.g. absence of nutrients, UV light, heat treatment, radiation, desiccation, high pressure, toxic chemicals) that vegetative cells would not survive, and their formation by Group I *C. botulinum* and *C. sporogenes* is a primary reason why these bacteria present a significant food safety and food spoilage problem. Significantly, strains of Group I *C. botulinum* and *C. sporogenes* form spores of very high heat resistance, and the “botulinum cook” has been adopted by the canning industry as the standard minimum heat treatment (121°C for 3 min) for low acid canned foods [1]. Under suitable conditions, the dormancy of bacterial spores is broken, and germination occurs. This is often initiated by a germinant receptor (GR) located in the spore inner membrane responding to nutrient germinants, and is followed by the release of dipicolinic acid and partial core hydration. Later, cortex-lytic enzymes degrade peptidoglycan in the spore cortex, enabling further core hydration and core expansion, and this results in the loss of spore resistance [24]. Germination involves pre-formed enzymes located in the dormant spore, and is followed by the initiation of metabolism and macromolecular synthesis, eventually leading to the emergence of a cell that is able to multiply. One approach that has interested microbiologists for many decades is to develop strategies to either prevent spore germination altogether and thereby prevent subsequent growth, or to germinate all the spores and then inactivate the emergent sensitive vegetative cells. Unfortunately the highly heterogeneous nature of spore germination (as observed for example with Group II *C. botulinum*) [25]–[27] has prevented the development of suitable processes. However, a greater understanding of spore germination in Group I *C. botulinum* and *C. sporogenes* may enable the development of novel intervention strategies to prevent or reduce disease and other adverse events such as food spoilage.

Spore germination in *Bacillus* species generally involves a GR located in the spore inner membrane. The GR is composed of three proteins (A, B and C) that are encoded in a tricistronic operon. The A and B proteins are integral membrane proteins, while the C proteins are lipoproteins [24], [28]. Spore germination in *Clostridium* species is not as extensively studied as that in *Bacillus* species, although recently significant advancements have been made with several clostridia including *C. perfringens*, *C. difficile* and *C. sordellii* [24], [29]–[43]. Spore germination in clostridia often proceeds more slowly than that in *Bacillus* species [1], and recent evidence suggests that although there are many similarities there are also a number of important differences in spore germination between clostridia and *Bacillus* species [24], [28], [29]. For example, spores of *Bacillus* species require a complete GR system to germinate effectively, and while this is also the case for some clostridia, spores of *C. difficile* are able to germinate effectively in the absence of what is classically understood as a GR. Such differences between clostridia probably reflect their wide genetic diversity [1], [24]. Spores of Group I *C. botulinum* and *C. sporogenes* germinate when specific germinant nutrients such as a combination of L-alanine and L-lactate (with less efficient germination in response to other amino acids and L-lactate or single amino acids [3], [44]) interact with a GR located in the clostridial spore inner membrane. GR operons are well conserved amongst strains of Group I *C. botulinum*. Group I *C. botulinum* type A strains ATCC3502, Hall, ATCC19397, Kyoto and NCTC2012 possess a pentacistronic GR operon (*gerXB-XA2-XB2-XC2-XB*), two tricistronic GR operons (*gerXA1-XB1-XC1* and *gerXA3-XB3-XC3*), and an orphan *gerXB* homologue [3], [45]. Gene clusters resembling the pentacistronic GR operon and *gerXA3-XB3-XC3* have been characterised in *C. sporogenes* strain NCIMB701792 and Group I *C. botulinum* type B strain NCTC7273, respectively [16], [46]. Strains of Group I *C. botulinum* type B (Okra) and F (Langeland) possess an additional tricistronic GR operon (*gerXC4-XA4-XB4*) [3], [45]. It is still not known, however, to which nutrient germinant(s) the various specific GRs are responding, or the relative importance of each of the GRs.

The purpose of the present study was to dissect, at the molecular level, spore germination in Group I *C. botulinum* strain ATCC3502 and in *C. sporogenes* strain ATCC15579. In particular, the key aims were to establish for each strain which of the multiple nutrient GRs was active in spore germination, and for the active nutrient GRs which nutrient germinant(s) they responded to. This was achieved using a combination of genomic, genetic and physiological approaches. The spore GRs in the two clostridia showed a number of interesting similarities, and this study provided further evidence of differences between spore germination in *Clostridium* and *Bacillus* species. Interestingly, subtle differences were also noted in spore germination between that in Group I *C. botulinum* strain ATCC3502 and that in *C. sporogenes* strain ATCC15579. This study has provided novel insights into spore germination in these two important clostridia.

## Results

### Involvement of L-lactate in amino acid induced germination in *C. botulinum and C. sporogenes*

**Figure 1:**
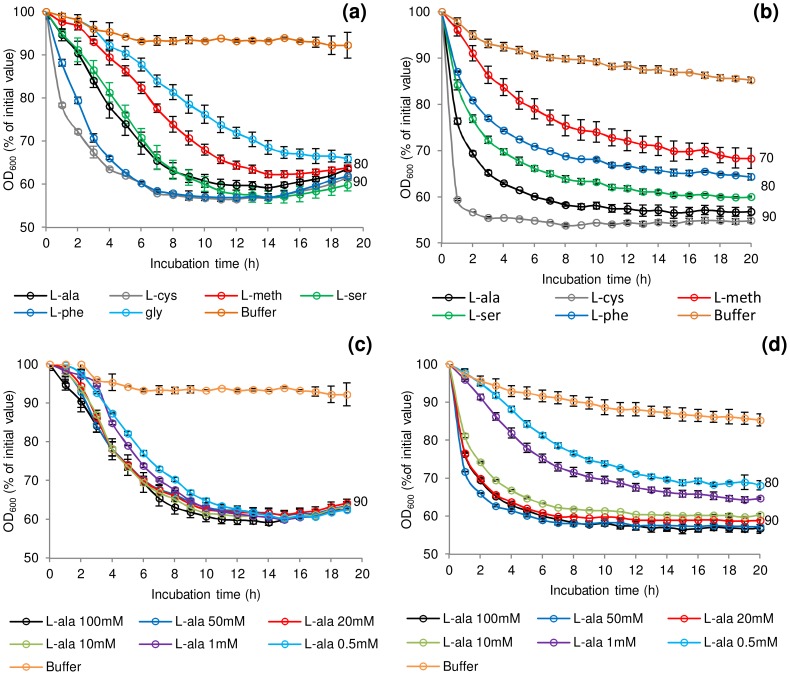
Rate of *C. botulinum* and *C. sporogenes* spore germination in the presence of selected amino acids. Effect of L-alanine, L-cysteine, L-methionine, L-serine, L-phenylalanine and glycine (*C. botulinum* only) at 100 mM on germination of (a) *C. botulinum* ATCC3502 and (b) *C. sporogenes* ATCC15579. The effect of increasing L-alanine concentrations (0.5 mM–100 mM) on germination was also tested on (c) *C. botulinum* ATCC3502 and (d) *C. sporogenes* ATCC15579. Tests were conducted in 20 mM Tris buffer, pH 7.4, L-lactate (50 mM) and NaHCO_3_ (50 mM) at 30°C. Buffer only controls contained 20 mM Tris, pH 7.4, L-lactate (50 mM) and NaHCO_3_ (50 mM). Data labels (right) refer to percentage germination observed by phase contrast microscopy at the end of the experiment. Error bars represent the standard deviation of 3 independent experiments.

A selection of amino acids at various concentrations (Table S1) were assessed for their individual effect on germination of spores of *C. botulinum* and *C. sporogenes*. The majority of the amino-acids tested have previously been reported to contribute as germinants or co-germinants for spores of *Clostridium* or *Bacillus* [1]. Initial tests showed that spore germination was similar under aerobic and anaerobic conditions (data not shown). This confirmed previous reports for *C. botulinum* and *C. sporogenes* [47], [48]. In the presence of Tris buffer (pH 7.4), L-lactate (50 mM) and NaHCO_3_ (50 mM) at 30°C the addition at 100 mM of either L-alanine or L-cysteine initiated spore germination in *C. botulinum* and *C. sporogenes*, although at differing rates (Figure 1a & 1b). L-lactate was not essential for L-alanine or L-cysteine stimulated germination and had no effect on rate or the overall extent of germination (data not shown). Three amino acids (L-methionine, L-serine, L-phenylalanine) each required L-lactate for inducement of germination in both species. The addition of L-lactate on its own failed to stimulate germination (<10% fall in OD_600_, equating to <1% germination). Spores of *C. botulinum*, but not those of *C. sporogenes*, were also germinated by glycine in combination with L-lactate (Figure 1a). L-cysteine combined with L-lactate produced the most rapid germination of *C. botulinum* spores (40% of initial OD_600_, approximately 90% germination after 6 h) and *C. sporogenes* spores (40% of initial OD_600_, approximately 90% germination after 4 h). *C. sporogenes* germination proceeded far more rapidly to completion with all the tested amino acids compared to *C. botulinum*. A number of other amino acids were tested, but failed to induce spore germination in either *C. botulinum* or *C. sporogenes*, both in the presence and absence of L-lactate (Table S1). For both species, the saturation concentration of the amino acids was 50–100 mM with l-lactate at 50 mM (Figure 1c & 1d). For simplicity, L-lactate was included in all subsequent germination studies. The optimum pH range was pH 6–8 for germination with most amino acids + l-lactate. However, spore germination was also observed at pH 10 in the presence of L-serine, and L-alanine + l-lactate. The rich microbiological growth medium, TY medium, was not optimum for spore germination, with less germination observed than in the defined system, although the addition of L-lactate did increase the rate and overall extent of germination in TY medium. Spore germination measured using the Bioscreen system correlated well with direct counts of phase-dark spores by phase-contrast microscopy (data not shown) with an OD_600_ fall of 40% correlating to >90% germination of spores.

### Spore production environment affects rate of germination

**Figure 2:**
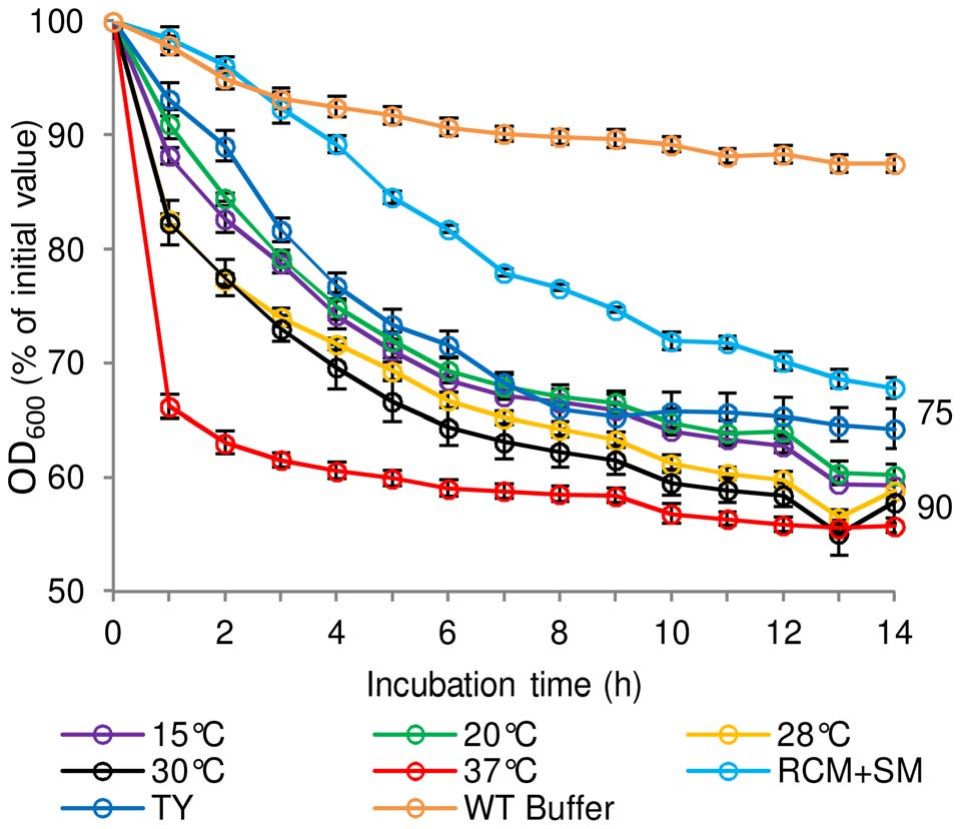
The effect of spore production environment on subsequent germination of *C. sporogenes* spores. For germination tests, spores were incubated in 20 mM Tris buffer, pH 7.4, L-alanine (100 mM), L-lactate (50 mM) and NaHCO_3_ (50 mM) at 30°C. Spores were produced in CMB at different temperatures; 15°C, 20°C, 28°C, 30°C, 37°C. Spores were also produced in TY broth at 37°C and on RCM+SM plates at 37°C. Spores produced at 37°C in CMB and incubated in 20 mM Tris, pH 7.4, L-lactate (50 mM) and NaHCO_3_ (50 mM) only, were included as a negative control (WT Buffer). Spore germination was confirmed by phase contrast microscopy. Data labels (right) refer to percentage germination observed by phase contrast microscopy at the end of the experiment. Error bars represent the standard deviation of 3 independent experiments.

The effect of sporulation medium and sporulation temperature on the subsequent germination properties of *C. sporogenes* spores was determined (Figure 2). Assessment of *C. botulinum* was precluded by frequent poor sporulation of this strain, which was less than 5% and 1% in Robertson's cooked meat broth (CMB) and TY respectively, compared to the optimum yield of 30% spores on RCM plus skimmed milk (RCM+SM) plates at 37°C. *C. sporogenes* spores were produced using either RCM+SM, CMB or TY broth at 37°C. Microscopic observations showed sporulation was notably lower (ca. 40%) using CMB and TY compared to RCM+SM. *C. sporogenes* spores were also produced at 15, 20, 28, 30, 37 and 42°C in CMB to evaluate the effect of temperature. Microscopic observations showed that sporulation was notably lower (ca.50%) at 15°C compared to 37°C in CMB, with sporulation not observed at 42°C. Germination of the spores was then evaluated in the presence of L-alanine + L-lactate (Figure 2). Interestingly, spores produced in CMB at 37°C germinated at a faster rate compared to spores produced in the other media at 37°C. Sporulation temperature also affected germination rates, with spores produced at 37°C germinating more rapidly than spores produced at other temperatures (Figure 2). Subsequently, all spore crops used in germination studies were produced at 37°C in CMB for *C. sporogenes* and on RCM+SM for *C. botulinum*.

### D-amino acids prevent L-amino acid induced germination

Unlike their L-isomers, D-alanine, D-cysteine, D-methionine, D-phenylalanine and D-serine all failed to trigger spore germination in either *C. sporogenes* or *C. botulinum*. Moreover, the D-amino acids prevented spore germination (defined as a <10% fall in OD_600_, equating to <1% germination observed microscopically) in *C. botulinum* and *C. sporogenes* induced by their equivalent L-amino acid (Table 1). To ascertain if specific D-amino acids could prevent germination by non-equivalent L-amino acids, D-serine was tested at a ten-fold excess of each of the five L-amino acids. Interestingly, D-serine prevented germination in *C. sporogenes* induced by L-cysteine, L-methionine, L-phenylalanine and L-serine, and to a lesser extent L-alanine. L-alanine and L-cysteine induced germination was only slightly affected by a ten-fold excess of D-serine in *C. botulinum*. However, when a 100-fold excess of D-alanine was added, spore germination in the presence of each of the five L-amino acids was prevented in *C. botulinum*. Finally, in order to assess whether the D-amino acid is acting specifically rather than being simply in excess of the L-amino acid germinant, we performed further tests using different germinants (L-alanine, 1 mM or L-serine, 20 mM) each combined with an excess (100 mM and 40 mM respectively) of the non-germinant L-valine. The addition of excess non-germinant in each experiment had no effect on final germination levels. However, the D-amino acids (in this case alanine or serine) continued to be inhibitory.

**Table 1:** Minimum D-amino acid concentration required to prevent* germination by its equivalent L-amino acid.

	D-amino acid concentration	
L-amino acid/concentration	*C. sporogenes*	*C. botulinum*
L-alanine/1 mM	10 mM	100 mM
L-cysteine/10 mM	100 mM	100 mM
L-serine/10 mM	20 mM	100 mM
L-phenylalanine/10 mM	20 mM	NT
L-methionine/10 mM	100 mM	100 mM

Tests were conducted in 20 mM Tris buffer, pH 7.4, L-amino acid (1–10 mM), D-amino acid (10–100 mM) + L-lactate (50 mM) and NaHCO_3_ (50 mM) at 30°C. NT = D-phenylalanine was not inhibitory at 20 mM and could not be tested at a higher concentration due to low solubility.

*Defined as a <10% fall in OD_600_, equating to <1% germination observed microscopically.

### Homologues of the *C. botulinum* nutrient germinant receptor operons in *C. sporogenes*

**Figure 3:**
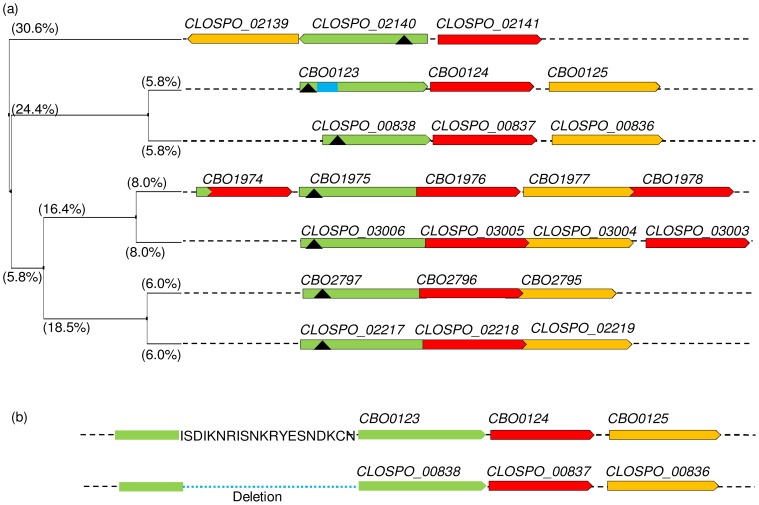
Alignment of *C. botulinum* and *C. sporogenes* germinant receptor proteins. Homologues of *C. botulinum* (strain ATCC3502) GRs were identified by BLASTp analyses using the draft un-assembled genome of *C. sporogenes* (strain ATCC15579). (a) Tree calculated (using Jalview [76]) from the pairwise sequence distances between GerXA only (determined from % sequence identities) of *C. sporogenes* (CLOSPO_number.) and *C. botulinum* (CBOnumber.) GRs, using the UPGMA algorithm [76]; average distances between GerXA (green) are shown on the branches. GerXB (red) and GerXC (yellow) are shown on the same tree (UPGMA produced identical-topology trees for each of the GerXB, GerXC proteins; distances not shown). (black triangle) Position of insertion sites of retargeted introns for mutations (in equivalent DNA sequence). Small green coloured region of CBO1974 represents a small protein fragment (CBO1973A), the coding sequence of which overlaps that of CBO1974, with homology to the C terminus of a GerXA protein. Blue square; 20 amino acid section that is deleted in its *C. sporogenes* homologue, CLOSPO_00838. (b) More detailed version of part of the above tree, showing the amino acid sequence encoded by a region in CBO0123 that is deleted from its *C. sporogenes* homologue, CLOSPO_00838.

Homologues of Group I *C. botulinum* strain ATCC3502 GR sub-units (*gerXA*, *gerXB*, *gerXC*) were identified by BLASTp analyses against a draft un-assembled genome of *C. sporogenes* strain ATCC15579 (Figure 3). Analysis showed that the *C. sporogenes* strain contains three tricistronic GR operons and one tetracistronic GR operon. In comparison *C. botulinum* ATCC3502 has two tricistronic GR operons and one pentacistronic GR operon. Each strain has an additional orphan *gerXB* subunit homologue. Alignment using Clustal Omega and using Jalview to produce a tree showing the average distance based on amino acid sequence identity (%), revealed homology between the GR operons (Figure 3). Each operon in *C. botulinum* had a closely related operon in *C. sporogenes* (from 11.6–16.0% difference in identity for each *gerXA*), while the CLOSPO_02140 *gerXA* is most distant with regards to sequence % identity. Transmembrane helix (TMH) prediction analysis showed that the *gerXA* sub-units of *C. botulinum* and *C. sporogenes* have between three and five TMHs, and the *gerXB* subunits have ten TMHs. The *gerXC* subunits were predicted to be lipoproteins and encode a signal peptide. Interestingly, more detailed sequence analysis of the pentacistronic operon of *C. botulinum* reveals that although the first gene of the operon, CBO1974, is a full-length member of the *gerXB* family, the 5′ end of its coding region is overlapped by a small (162 bp) region of a *gerXA* gene, annotated as CBO1973A. Comparative analysis between *C. botulinum* and *C. sporogenes* ger homologues also revealed that the *gerXA* gene, CLOSPO_00838 lacks an uninterrupted region encoding 20 amino acid residues which map to residues 74–93 of the predicted translation for CBO0123 (Figure 3b). A database search shows that this deletion (with respect to *C. botulinum* strain ATCC3502) is not confined to *C. sporogenes* strain ATCC15579, but can be found in 10 of a total of 19 GerA peptide sequences from proteomes of *C. sporogenes* (2), Group I *C. botulinum* (4) and Group III *C. botulinum* (4) (data not shown). Similar deletions were not found in any Group II *C. botulinum* GerA peptides. The function of this apparently conserved region remains unknown.

### Mutation of putative germinant receptors

To characterise the functionality of the putatively identified germination GRs, a series of single (*C. botulinum* and *C. sporogenes*), and triple (*C. sporogenes*) insertion mutants were constructed. Furthermore, a *C. sporogenes* quadruple insertional knockout GR mutant (*gerXA* ^4−^) was also generated. The current insertional knockout system does not allow multiple insertion selection, as following one insertion the single mutant is then erythromycin resistant. However, the mutant generation system was shown to be highly efficient, which negated the need for a different antibiotic selection in the multiple insertion mutants. All insertion events were tested by PCR which confirmed chromosomal integration of the intron (Figure S1). PCR using gene specific and intron specific primers confirmed insertion of the intron into the GR gene; this was further confirmed by PCR with gene specific primers flanking the insertion site producing a ∼2 kb product (Figure S1). Insertion events were verified by Southern hybridization using an intron specific probe which confirmed the correct number of insertion events in all the constructed mutants (Figure S1c).

### *C. botulinum* requires two tri-cistronic receptors for germination

**Figure 4:**
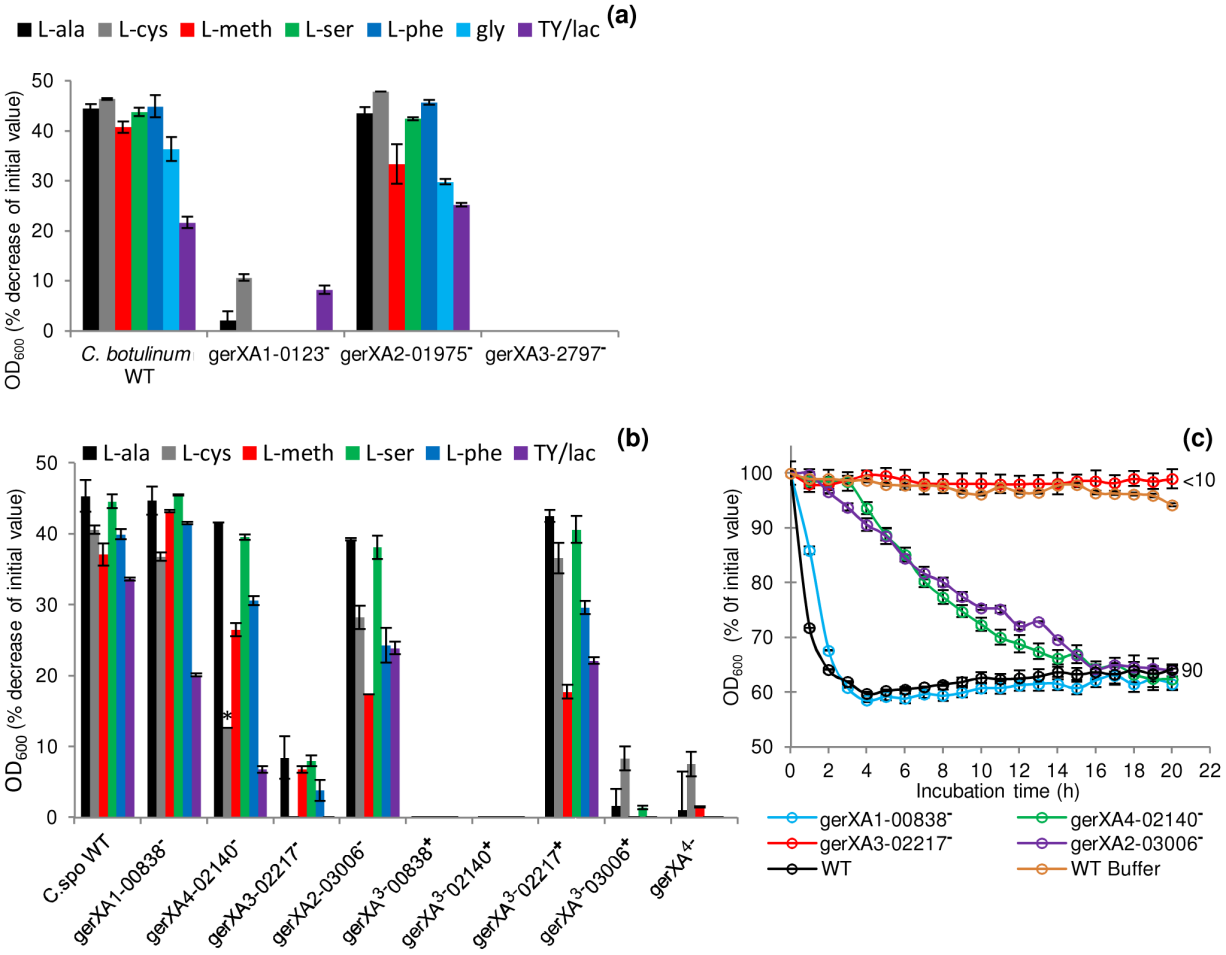
Mutation of specific receptors precludes amino acid stimulated germination. Spores were incubated in 20 mM Tris buffer, pH 7.4, amino acid (100 mM) + L-lactate (50 mM) and NaHCO_3_ (50 mM) at 30°C for 20 hours with L-alanine, L-cysteine, L-methionine, L-serine, L-phenylalanine, glycine (*C. botulinum* only) or in TY + L-lactate (50 mM). (a) *C. botulinum* single insertional knockout mutants and wild type spore germination. (b) *C. sporogenes* single insertional knockout mutants, triple insertional knockout mutants, quadruple insertional knockout GR mutant and wild type spore germination. * L-cysteine is a relatively insoluble amino acid and precipitates out of solution after 2 hours. Due to the 4 hour delay in germination of the mutant *gerXA4*-02140^−^ cysteine precipitation caused OD_600_ readings to be unrepresentative and therefore germination was confirmed by microscopy. (c) Alanine induced germination rates of single insertional knockout GR mutants for *C. sporogenes* were determined using spores generated from the wild type (*C. sporogenes* ATCC15579) and mutants *gerXA1*-00838^−^, *gerXA4*-02140^−^, *gerXA3*-02217^−^, *gerXA2*-03006^−^. WT spores incubated in buffer only (see above), were included as a negative control (WT Buffer). Data labels (right) refer to percentage germination observed by phase contrast microscopy at the end of the experiment. Error bars in (a–c) represent the standard deviation of 3 independent experiments. Spore germination was confirmed by phase contrast microscopy.

**Figure 5:**
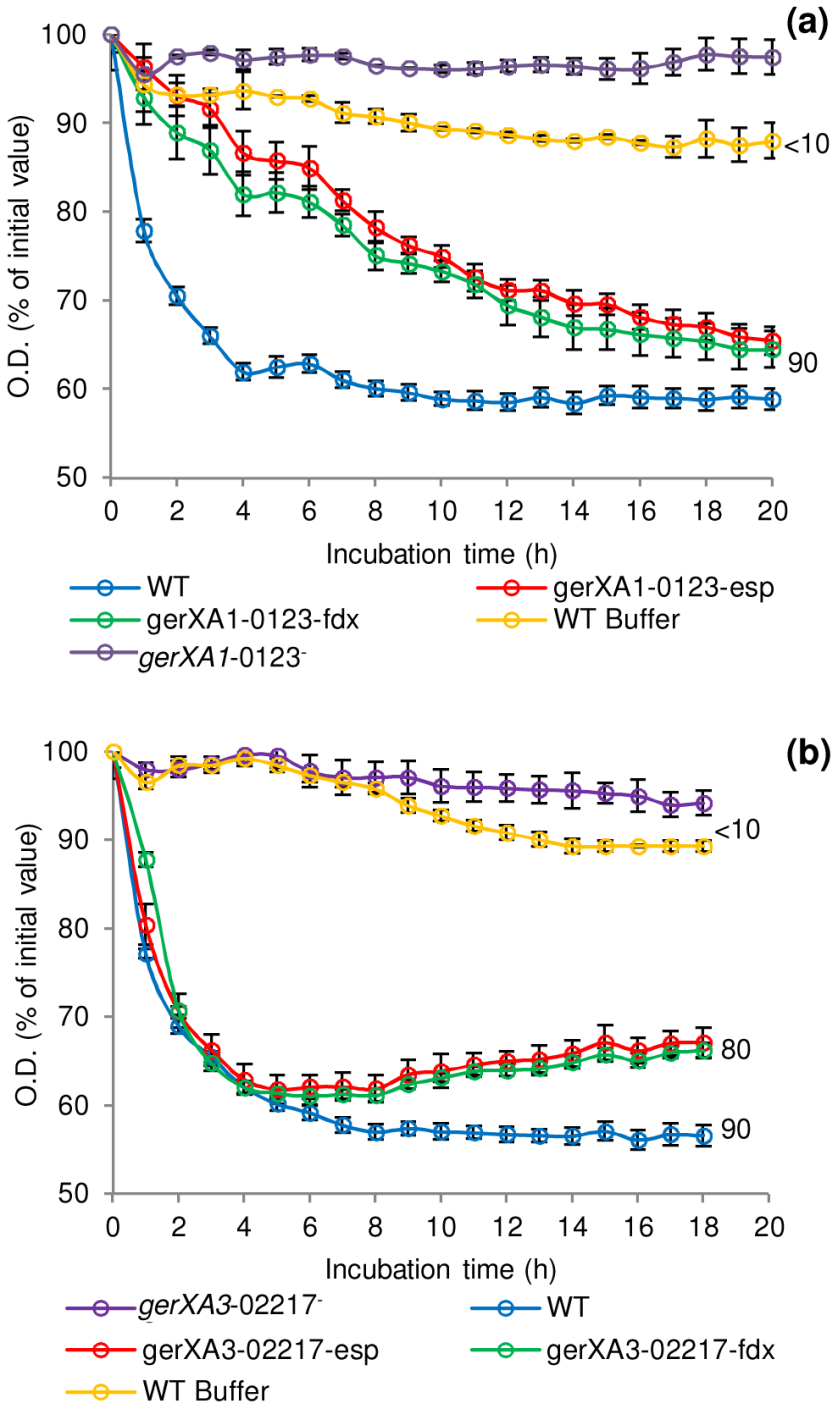
Germination rates of complemented GR mutants for *C. botulinum* and *C. sporogenes*. Spores were incubated in 20 mM Tris buffer, pH 7.4, amino acid (100 mM) + L-lactate (50 mM) + NaHCO_3_ (50 mM) at 30°C with L-cysteine (*C. botulinum*), L-alanine (*C. sporogenes*). (a) *C. botulinum* mutant *gerXA1*-0123^−^ complemented with plasmid pMTL8315esp (*gerXA1*-0123^−^esp) or plasmid pMTL8315fdx (*gerXA1*-0123^−^fdx). (b) *C. sporogenes* mutant *gerXA3*-02217^−^ complemented with plasmid pMTL8315esp (*gerXA3*-02217^−^esp) or plasmid pMTL8315fdx (*gerXA3*-02217^−^fdx). There were two negative controls. Firstly, the uncomplemented mutant (*gerXA1*-0123^−^ or *gerXA3*-02217^−^), secondly WT spores incubated in 20 mM Tris buffer (pH 7.4) + L-lactate (50 mM) + NaHCO_3_ (50 mM) only (WT Buffer). Spore germination was confirmed by phase contrast microscopy. Error bars represent the standard deviation of 3 independent experiments. Data labels (right) refer to percentage germination observed by phase contrast microscopy at the end of the experiment.

To characterise the GRs and to identify their cognate germinants, single insertional knockout mutants (*gerXA1*-0123^−^, *gerXA2*-1975^−^, *gerXA3*-2797^−^) were created for each of the identified GR operons in *C. botulinum*. Spores generated from these mutants were then analysed for amino acid stimulated germination using L-alanine, L-cysteine, L-methionine, L-serine, L-phenylalanine or glycine, all in the presence of L-lactate (Figure 4a). The OD_600_ of wild-type spores decreased (∼40%) indicating efficient and complete spore germination in the presence of each amino acid. There was a ∼20% decrease in OD_600_ with TY medium + L-lactate. In contrast, the *gerXA3*-2797^−^ mutant failed to germinate with any of the amino acids tested even after 20 h of exposure (<1% germination observed microscopically). Moreover, germination was not observed with a nutrient rich broth (TY + L-lactate) suggesting that CBO2797 is essential for amino acid stimulated germination. The mutant *gerXA1*-0123^−^ also failed to germinate to the same extent as the wild type, with a small decrease (<10%) in OD_600_ observed with L-cysteine and with nutrient rich broth (TY + L-lactate). In neither case could spore germination be observed microscopically. These results suggest that CBO0123 is also essential for amino acid induced germination. Interestingly, the *gerXA2*-1975^−^ mutant showed similar germination patterns to those of WT spores (Figure 4a). Complementation was performed by two different approaches; using plasmid pMTL83151esp, which relies on the putative native promoter of the gene, or using pMTL83151fdx which includes the powerful promoter P*_fdx_* of the ferredoxin gene (*fdx*) from *C. sporogenes*. Complementation was successful for one of the two GerXAs observed to be important for nutrient-induced germination. Introduction of the receptor CBO0123-CBO0124-CBO0125 complementation vector (pMTL83151esp or pMTL83151fdx) successfully restored germination to the mutant *gerXA1*-0123^−^, albeit at a different rate compared to that of the wild type (Figure 5a). Introduction of the GR CBO2797-CBO2796-CBO2795 complementation vector (pMTL83151esp or pMTL83151fdx) drastically reduced sporulation efficiency, giving insufficient spores to allow assessment of the germination phenotype.

**Figure 6:**
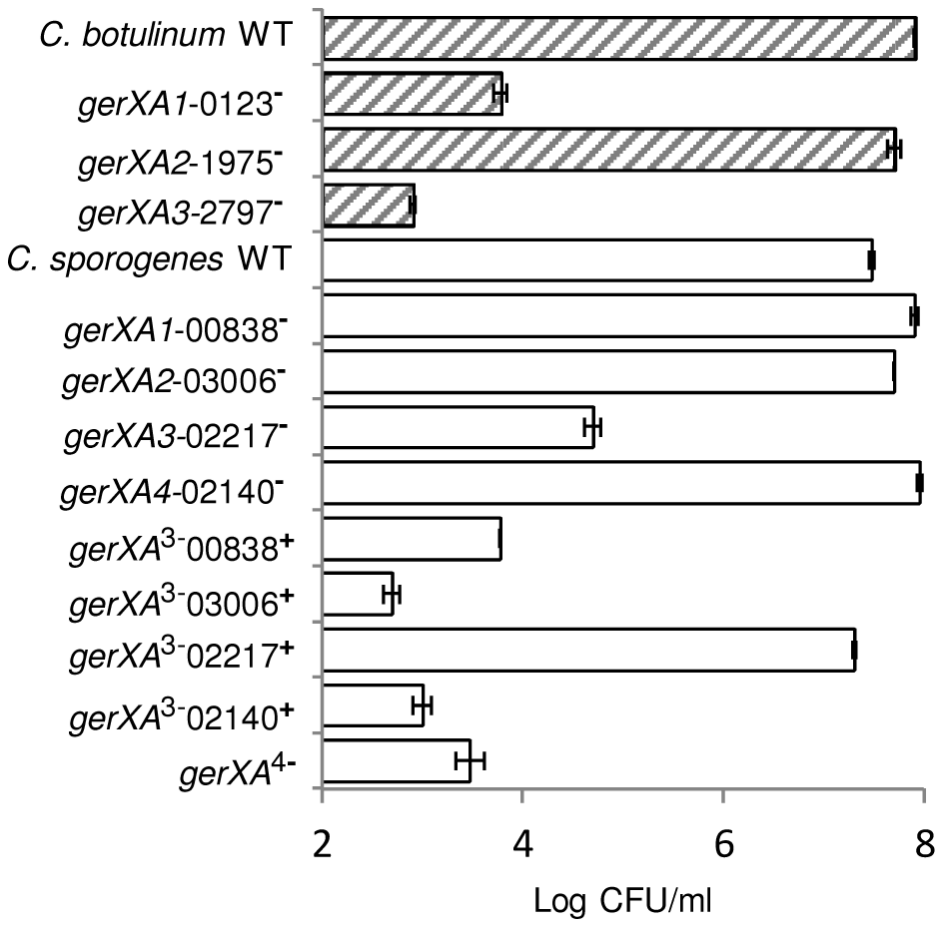
Capacity of WT or mutant spores to germinate and form colonies. *C. botulinum* and *C. sporogenes* wild type and mutant spore suspensions were enumerated using a haemocytometer and adjusted to a final concentration of ∼1×10^8^ spores/ml. Spores were heat activated (80°C, 15 min), serially diluted in 0.85% saline, and plated in triplicate on to TY agar before anaerobic incubation (37°C, 48 hrs), after which colonies were enumerated. Data presented represent the mean log_10_ colony-forming units/ml from triplicate plates, with error bars representing the standard deviation of the mean.

Finally, these results were further supported by examining the number of colonies formed on TY plates after incubation for 2 days at 37°C. All spore crops were adjusted to a final concentration of ∼1×10^8^ spores/ml, serially diluted, and plated on to TY agar. Single mutant *gerXA2*-1975^−^ showed comparable numbers of colonies to the wild-type. In contrast, mutants *gerXA1*-0123^−^ and *gerXA3*-2797^−^ exhibited a greatly reduced colony forming efficiency (Figure 6).

### A single tri-cistronic receptor in *C. sporogenes* is essential for germination

To characterise the *C. sporogenes* GRs and to identify their cognate germinants, single insertional knockout mutants (*gerXA1*-00838^−^, *gerXA2*-03006^−^, *gerXA3*-02217^−^, *gerXA4*-02140^−^) were created for each of the identified GR operons. The OD_600_ of wild-type spores decreased (∼40%) indicating efficient and complete spore germination (Figure 4b). Mutant *gerXA3*-02217^−^ failed to germinate (<10% fall in OD_600_; spore germination not observed microscopically) with any of the amino acids tested after 20 h of exposure (Figure 4b & 4c). Spore germination was not observed with a nutrient rich broth (TY + L-lactate) demonstrating that CLOSPO_02217 is required for amino acid stimulated germination. Germination of mutant *gerXA2*-03006^−^ showed an initial delay of one hour compared to the wild type with all the amino acids tested (e.g. Figure 4c). However, following this interval, germination proceeded to the same extent, albeit at a slower rate compared to the wild type (Figure 4b & 4c). Similarly, *gerXA4*-02140^−^ had an initial germination postponement of four hours and then proceeded to germinate fully, but also at a slower rate. No discernible phenotype was observed following insertional inactivation of the *gerXA1* (mutant *gerXA1*-00838^−^) GR compared to the WT (Figure 4b & 4c).

To further understand the function of the individual GRs in germination, triple insertional knockout GerXA mutants (*gerXA* ^3−^00838^+^, *gerXA* ^3−^02140^+^, *gerXA* ^3−^02217^+^, *gerXA* ^3−^03006^+^) and a quadruple insertional knockout GerXA mutant *gerXA* ^4−^ were created. Mutation of three GerXAs resulted in one remaining potentially functional GerXA so any possible interaction between GerXAs is excluded and so enables dissection of the specific germinant recognised. As anticipated, spores from the quadruple insertional knockout mutant *gerXA* ^4−^ failed to germinate (<10% fall in OD_600_, germination not observed microscopically) with any of the amino acids or in nutrient rich broth (TY + L-lactate) (Figure 4b). Furthermore, the three triple mutants *gerXA* ^3−^00838^+^, *gerXA* ^3−^02140^+^, and *gerXA* ^3−^03006^+^ also failed to germinate with the amino acid systems or in nutrient rich broth (TY + L-lactate). Mutant *gerXA* ^3−^02217^+^ which has only a single active GerXA present (CLOSPO_02217), displayed comparable germination rates to the wild type with all the amino acids tested and the nutrient rich broth (TY + L-lactate) (Figure 4b). Complementation of all the GerXAs further confirmed these findings. Particularly important was the introduction of the GR CLOSPO_02217- CLOSPO_02218- CLOSPO_02219 complementation vectors (pMTL83151esp or pMTL83151fdx), as these fully restored germination to the mutant *gerXA3*-02217^−^ to WT levels (Figure 5b).

Finally, the number of colonies formed on TY plates after incubation for 2 days at 37°C was determined. All spore crops were adjusted to a final concentration of ∼1×10^8^ spores/ml, serially diluted, and plated on to TY agar. The wild-type and single insertion mutants *gerXA1*-00838^−^, *gerXA4*-02140^−^ and *gerXA2*-03006^−^ formed comparable numbers of colonies. In contrast, mutant *gerXA3*-02217^−^ showed a greatly reduced colony forming efficiency (Figure 6). Triple mutants *gerXA* ^3−^00838^+^, *gerXA* ^3−^02140^+^, *gerXA* ^3−^03006^+^ and the quadruple insertional knockout mutant *gerXA* ^4−^ exhibited a significantly reduced colony forming efficiency compared to the WT. Importantly, the triple mutant *gerXA* ^3−^02217^+^, revealed comparable numbers of colonies to the wild type (Figure 6).

## Discussion

One important feature that has contributed to the success of botulinum neurotoxin-forming clostridia, and all other clostridia, is their ability to form highly resistant endospores. Under suitable conditions the spores germinate with associated loss in resistance properties, and cell multiplication recommences. Spore germination occurs through a number of steps that are poorly understood in clostridia. The present study has identified which nutrient germinants are able to stimulate spore germination in Group I *C. botulinum* ATCC3502 and in *C. sporogenes* ATCC15579, has for the first time identified which of the individual GRs are responding to these nutrient germinants. A survey of the available genomes of Group I *C. botulinum* and its close relative, *C. sporogenes* reveals that although the general trend is to possess three or four operons encoding spore GR proteins, the fine detail of this organisation varies. The gerXC subunits were predicted to be lipoproteins and encode a signal peptide. In *Bacillus*, a lipobox consensus sequence (GCX) has been recognised within the first 30 residues of the GRs C subunits, where the cysteine in this motif is diacylglycerylated to facilitate cleavage of the signal peptide. In *C. botulinum* this lipobox was observed in *gerXC* gene CBO0125 and in two *C. sporogenes gerXC* genes CLOSPO_00836 and CLOSPO_02139. However, diacylglycerol addition does not appear to be essential for function [29]. In addition to the existence of orphan *ger* genes (such as CBO2300 in Group I *C. botulinum* ATCC3502), there is evidence of genetic recombination at these loci, most obviously the presence of multiple *gerXB* genes in the pentacistronic operon of Group I *C. botulinum* ATCC3502 (CBO1974-1978), and in its tetracistronic equivalent in *C. sporogenes* ATCC15579 (CLOSPO_03006-03003). Less obvious evidence includes the small fragment of a *gerXA* gene apparently inserted into the beginning of the first ‘extra’ *gerXB* gene of the Group I *C. botulinum* ATCC3502 pentacistronic operon, and the extra (or deleted) 20 codons discovered by comparison of the coding sequences of CBO0123 and CLOSPO_00838. Site-directed mutagenesis studies will be required to determine the functional status of these genetic differences.

Spore germination in Group I C. *botulinum* ATCC3502 and *C. sporogenes* ATCC15579 was triggered by a variety of amino acids, often in combination with L-lactate. L-phenylalanine + L-lactate and L-cysteine + L-lactate were the most effective germinants for *C. botulinum*, while L-cysteine + L-lactate were the most effective germinant for *C. sporogenes*. L-lactate had no discernible effect on spore germination in the presence of L-alanine or L-cysteine, but was essential for germination induced by L-methionine, L-serine or L-phenylalanine. Previous studies have reported a variable effect of L-lactate on amino acid induced spore germination in Group I *C. botulinum* and *C. sporogenes* [1], [44], [46]. Germination of Group I C. *botulinum* and *C. sporogenes* with L-serine and glycine has been reported previously [44], [49], while germination induced by L-methionine + L-lactate in Group I *C. botulinum* appears to be a novel finding. L-methionine triggered germination has been previously reported in *C. sporogenes* [49], *B. anthracis* [50] and *C. tetani* [51]. Germination in *C. botulinum* and *C. sporogenes* was also induced by L-phenylalanine + L-lactate, as reported previously in *C. bifermentans* [52] and *C. sordellii* [30], whereas the GR GerI in *B. cereus* interacts with L-phenylalanine in combination with inosine [53]. Moreover, L-phenylalanine stimulation of *C. botulinum* germination was more effective than that obtained with L-alanine. It may be that L-phenylalanine and L-lactate interact before interacting with the GR or that L-lactate and L-phenylalanine may directly affect the GR together or sequentially. Any effect is unlikely to be due to the hydrophobic nature of L-phenylalanine, as L-alanine and L-cysteine also have polar side chains and induce germination efficiently. In the present study, germination was more rapid when induced by single amino acids with L-lactate than in the nutrient rich medium TY; a similar observation has been made for Group II *C. botulinum* [54]. However, the addition of L-lactate to TY increased the germination rate significantly.

The production of spore crops is usually performed under conditions that maximise the spore yield [55], [56]. However, mounting evidence now suggests that sporulation conditions may have a direct effect on germination efficiency in *Bacillus* [55]–[57]. In the present study, a greater yield of *C. sporogenes* spores was achieved on RCM+SM plates compared with CMB and TY broths. However, the germination rate was initially more rapid with spores produced in CMB, albeit all spore crops achieved a similar extent of germination after 16 h. *B. subtilis* spores produced in a liquid medium germinated more readily than spores produced on plates [58]. Similarly, germination of *B. subtilis* spores with dodecylamine was also highly dependent on the method used for spore preparation [59]. Although the present study shows that media composition for sporulation does have an impact on germination, the reasons for these findings remain unclear. The effect of sporulation temperature in CMB on the yield of spores and their subsequent germination was also assessed. A greater number of spores were formed at 37°C than at lower temperatures, and they also germinated more readily. Thus, on this occasion spore yield and spore germination were positively correlated. For Group II *C. botulinum*, sporulation temperature affected spore yield and fatty acid content, but not heat resistance or germination [60]. For *B. subtilis*, sporulation temperature affected resistance to wet heat and spore coat protein levels [61]. Certainly, for *C. sporogenes* it is apparent that sporulation conditions have a direct effect on subsequent germination with the selected amino acids. It remains to be established whether this effect is due to the number and/or state of GRs, or to as yet unknown proteins that are involved in the germination pathway. However, it is clear from these results that sporulation conditions should be considered, especially when *Clostridium* studies in the food industry are to be performed to evaluate processing strategies, as these typically use spores produced under conditions where the yield has been maximised.

In the present study, D-amino acids failed to trigger spore germination and also prevented germination induced by their respective L-amino acid, as reported previously for other strains of Group I *C. botulinum* and *C. sporogenes* [44], [47], [48], [62]–[64]. It is noted that D-alanine was previously reported to be a competitive inhibitor of L-alanine induced germination in *C. sporogenes* [48]. However, D-alanine did not prevent germination of spores of Group II *C. botulinum* types B, E and F in L-alanine + l-lactate + NaHCO_3_ (pH 7·0) when added at ten-times the concentration of l-alanine [54]. Interestingly, in the present study, D-serine prevented germination induced by L-amino acids in *C. sporogenes*, and D-alanine prevented germination induced by L-amino acids in *C. botulinum*. These observations are consistent with those made by Montville *et al.* (1985), who reported that L-cysteine triggered germination was inhibited by D-alanine as well as by D-cysteine, and that L-alanine-triggered germination was inhibited by D-cysteine as well as by D-alanine in Group I *C. botulinum* strains B-aphis and Ba410 [64]. Montville *et al.* suggested that alanine and cysteine shared a common germinant binding site in spores of these two strains [64]. However, kinetic studies (e.g. [43]) are required to establish if the position is the same for Group I *C. botulinum* ATCC3502 and *C. sporogenes* ATCC15579. Studies with *B. megaterium* and *B. subtilis* suggest that the B-protein subunit of the GR presents the site for the receptor-ligand binding [65], [66], and although no evidence is presently available, the position may be similar in *C. botulinum* and *C. sporogenes*. Furthermore, *C. botulinum* (ATCC3502) and *C. sporogenes* (ATCC15579) both contain five putative alanine racemase genes. Alanine racemase is able to convert the germinant L-alanine into inhibitory D-alanine in *B. cereus* [67]. However, despite these clostridia containing five putative racemase genes, germination of Group I *botulinum* spores appeared not to be influenced by l-alanine racemase activity [44].

Molecular dissection of spore germination in Group I *C. botulinum* strain ATCC3502 demonstrated that two GerXAs were required for amino acid stimulated germination. The interruption of either gene CBO0123 or CBO2797 (mutants gerXA1-0123^−^ and gerXA3-2797^−^) resulted in no observable germination. Thus, it has been shown that for amino acid stimulated germination there is a minimum requirement for the GerXAs produced by these two GRs, and while the product of gene CBO1975 appears to be inactive, it cannot be ruled out that the other products (GerXB and GerXC) of this operon are functional. The requirement for two GRs for germination has been previously reported in *B. subtilis* [68]. In this bacterium the GRs GerB and GerK interact and responded to a cocktail of L-asparagine, D-glucose, D-fructose, and K^+^ (AGFK) [68]. The *B. anthracis* GRs GerK and GerL also act cooperatively with alanine to stimulate the germination pathway [69]. Importantly, they can also act individually and initiate germination with proline and methionine (GerK) or serine and valine (GerL) as cogerminants in conjunction with inosine [69]. It is presently unclear how pairs of GRs come together to induce germination; potential hypotheses include: (i) one GR of the pair is involved in the binding of the germinant and the second GR is involved in a signalling capacity; (ii) both GRs together may be required to form the germinant binding site; (iii) one GR may physically stabilise the receptor that receives the germinant. What is clear is that more evidence is required to characterise the individual role of each GR in germinant recognition. In the present study, single *C. botulinum* GRs failed to induce germination, either with single or with combinations of amino acids, or with components of a rich growth medium. Complementation restored wild type levels of germination to the mutant gerXA1-0123^−^, albeit at a slower rate compared to that of the wild type. However restoration of germination efficiency could not be assessed for the complementation mutant gerXA3-2797^−^ due to its poor sporulation efficiency. The failure of plasmid complemented mutants to regain wild type sporulation levels has been reported previously in *C. perfringens* [70]. Moreover, the use of multicopy plasmids can fail to restore the phenotype to wild type levels in clostridia [71]. It may be that in this instance inappropriately elevated levels of the GR proteins in the complementation mutant gerXA3-2797^−^ diminished sporulation efficiency. Undoubtedly, until techniques become available for stable integration of a single chromosomal copy of the complementing DNA, complementation studies in clostridia will remain challenging. Group I *C. botulinum* ATCC3502 also has a pentacistronic putative GR. This third GR, CBO1975-1977 (*gerXA2-XB2-XC2*), is unusual as it is flanked by two additional *gerXB* genes (CBO1974 and CBO1978) and is closely related to a GR gene cluster characterized in *C. sporogenes* ATCC15579 (this work). Furthermore, upstream of CBO1974 there is a pseudogene, CBO1973A with a disrupted ORF which would encode the C-terminal region of a GerA protein. This pseudogene overlaps the putative start of CBO1974 (*gerXB*). Insertion mutant (*gerXA2*-1975^−^) showed no attributable phenotype, with a similar germination pattern to the wild type strain. The pseudogene CBO1973A may hint at the possibility of recombinational events that have occurred at this locus that may have disrupted the normal control regions for correct GR expression. It cannot be ruled out that germination may be stimulated by suitable environmental stimuli that are not found in the nutrient rich medium, TY broth or any of the specific germinants tested in this work.

*C. sporogenes* is often regarded as the non-toxigenic equivalent of Group I *C. botulinum* [2], [19]. Comparisons of *C. botulinum* and *C. sporogenes* are important if *C. sporogenes* is to be used as a valid surrogate model in spore germination and other studies. There are four genes encoding GerXAs in *C. sporogenes* ATCC15579, and only one of these (CLOSPO_02217) was essential for amino acid induced germination. Two other GerXA proteins (products of CLOSPO_02140 and CLOSPO_03006) increased the rate of germination, providing that the product of CLOSPO_02217 was also present. Therefore, one GR (CLOSPO_02217-02219; and at least CLOSPO_02217) is required for amino acid stimulated spore germination. The remaining three GerXAs were not essential, but it cannot be ruled out that other products (GerXB and GerXC) of these operons may be functional and act synergistically with the CLOSPO_02217-02219 GR. Indeed, preliminary proteomics data from purified spores reveals that the *gerXC* gene CLOSPO_00836 is translated into a protein (data not shown). Although in *Bacillus* all subunits of GRs are required for a response to amino acids, the function of each individual subunit still remains to be elucidated [72]. The finding that, in *C. sporogenes* ATCC15579, some or all of the proteins from two GRs (CLOSPO_02139-02141 and CLOSPO_03003-03006) contribute to increase the rate of spore germination induced by a third GR (CLOSPO_02217-02219; or at least CLOSPO_02217) implies a close interaction between GR proteins. Different GRs may interact directly (and/or compete) with each other [73] or possibly one GR may facilitate access of the germinant to another GR. Although these two GRs were not stimulated by individual or a combination of amino acids, or by components of a rich microbiological growth medium, it is possible that they may individually respond to some other as yet unknown germinant. Interestingly, when the wild-type and various mutants containing an active CLOSPO_02217 were plated out on a rich growth medium recovery was complete (∼10^7^ CFU/ml), while in mutants where CLOSPO_02217 was insertionally inactivated (including in the quadruple *gerXA* ^4−^ mutant) the number of colonies recovered was significantly lower (∼10^3^–10^4^ CFU/ml). The recovery, at a very low frequency, of any spores in the absence of CLOSPO_02217 (including in the quadruple *gerXA* ^4−^ mutant) may be due to an alternative low efficiency receptor system distinct from the *ger* family, or perhaps to stochastic effects (e.g. cortex-lytic enzymes or ion/water channels). Similar observations have been made in *Bacillus subtilis* [74]. It is perhaps not surprising that this GR (CLOSPO_02217-02219) was involved in germination with the selected amino acids as it shares >85% homology with the functional and now characterised *C. botulinum* GR (CBO2795-2797). It is interesting that this GerXA can operate independently of any other GerXA, unlike in *C. botulinum*. One hypothesis is that the short deletion in *gerXA* of CLOSPO_00838 (when compared to its active *C. botulinum* homologue, CBO0123) is associated with loss of function. This mutational event in *C. sporogenes* may have brought about an evolutionary pressure which has allowed adaption of this organism to survive with only a single functional GR operon (or at least a single functional GerXA). Certainly the germination mechanism of *C. sporogenes* ATCC15579 is different to that of *C. botulinum* ATCC3502. Alignment of putative GRs with known functioning GRs appears to be problematic, as at present this is done over the whole protein in the absence of detailed knowledge of the functionality of the putative GR. However, based on comparison of the whole proteins, the GerXA of homologous GR proteins CBO2795-2797/CLOSPO_02217-02219 are functionally active, while no discernible function could be identified for the GerXA of GR proteins CBO1974-1978/CLOSPO_03003-03006. The GerXAs of other GR proteins were either essential (in *C. botulinum* ATCC3502) or contribute to the rate (in *C. sporogenes* ATCC15579). The results also call into the question the use of *C. sporogenes* as a suitable substitute for *C. botulinum* with regards to germination rates and germination substrates. More work is required to fully understand the role of each GR in clostridia and indeed why some species contain multiple GR operons and others can function with just one. The testing of additional strains would seem to be appropriate.

The long term aim is that as more is understood of the complex germination systems in clostridia, it may be possible to devise specific strategies to disrupt this process. This would be of great benefit to help control pathogenic clostridia, for example in the food industry, and might also help to control spore-disseminated nosocomial infections such as those caused by *C. difficile*.

## Materials and Methods

### Receptor identification and alignment

Homologues of *C. botulinum* ATCC3502 GR sub-units (*gerXA, gerXB, gerXC*) were identified by BLASTp analyses against a draft un-assembled genome of *C. sporogenes* strain ATCC15579. Alignment of *C. sporogenes* receptors with *C. botulinum* was performed using Clustal Omega [75] and Jalview [76] was utilised to produce a tree showing the average distance using % identity. Protein domain analysis was performed using Pfam [77]. Transmembrane helix prediction analysis of the GR sub-units was implemented using TMHMM [78].

### Bacterial strains and growth conditions

Proteolytic *C. botulinum* strain ATCC3502 (neurotoxin subtype A1) and *C. sporogenes* ATCC15579 were grown anaerobically at 37°C in tryptone-yeast medium (TY). The *Escherichia coli* strain Top10 (Invitrogen) was used for plasmid maintenance and the *E. coli* strain CA434 [79] was used for conjugal transfer. Both strains of *E. coli* were grown aerobically in Luria-Bertani medium (LB) at 37°C. Where appropriate, growth medium was supplemented with antibiotics at the following final concentrations; ampicillin 100 µg/ml, chloramphenicol 25 µg/ml, cycloserine 250 µg/ml, thiamphenicol 15 µg/ml, erythromycin 500 µg/ml (*E. coli*), 20 µg/ml (*C. botulinum*) 2.5 µg/ml (*C. sporogenes*), and the chromogenic substrate 5-bromo-4-chloro-3-indolyl-β-D-galactopyranoside (X-Gal) 80 µg/ml. All bacterial media supplements were purchased from Sigma. Spores of *C. botulinum* and *C. sporogenes* strains were prepared in TY, Reinforced Clostridial Medium plus skimmed milk (RCM+SM) or Robertson's cooked meat broth (CMB) (Southern Group Laboratories) and incubated at 15, 20, 28, 30, 37 or 42°C for a period of 10 days. Spores were cleaned and stored as described previously [54].

### PCR, cloning and Southern hybridisation

Constructed mutants and plasmids utilised in this study are presented in Table 2. Primers used for verification of successful insertion events and the Southern blot probes are listed in Table S2 (supplementary material). PCR experiments were carried out using Phusion High-Fidelity PCR Master Mix with GC Buffer kit (Thermo Fisher). Plasmid isolation and PCR purification was performed using the Wizard *Plus* SV Minipreps DNA Purification System and the Wizard SV Gel and PCR Clean-Up System (Promega) respectively, as described in the provided Technical Manual. Chromosomal DNA isolation from suspected mutants were prepared as previously described [16]. Restriction endonucleases and T4 DNA ligase were purchased from New England BioLabs and used according to the manufacturer's instructions. Southern hybridisation was performed to confirm the correct number of insertion events had occurred. The hybridisation probe was constructed by PCR to target the inserted intron using the primers Erm-F and Erm-R (Table S2). Genomic DNA (1 µg) was digested overnight with *Hin*dIII restriction enzyme and the fragments separated on a 1% agarose gel. Southern blot analysis was performed with ECL detection using a commercial kit (Amersham ECL Direct Nucleic Acid Labelling and Detection System) according to the manufacturer's instructions.

**Table 2:** Constructed mutants and plasmids utilised in this study.

Strain	CDS annotation	Locus	Insertion site	Mutant	Description
***C. botulinum*** ** (ATCC3502)**	*gerXA1*	CBO0123	42s	*gerXA1*-0123^−^	Single insertional knockout
	*gerXA2*	CBO1975	56s	*gerXA2*-1975^−^	Single insertional knockout
	*gerXA3*	CBO2797	1344a	*gerXA3-*2797^−^	Single insertional knockout
***C. sporogenes*** ** (ATCC15579)**	*gerXA1*	CLOSPO_00838	175s	*gerXA1*-00838^−^	Single insertional knockout
	*gerXA2*	CLOSPO_03006	795s	*gerXA2*-03006^−^	Single insertional knockout
	*gerXA3*	CLOSPO_02217	61s	*gerXA3*-02217^−^	Single insertional knockout
	*gerXA4*	CLOSPO_02140	601s	*gerXA4*-02140^−^	Single insertional knockout
	*gerXA1,*	CLOSPO_00838,	175s,	*gerXA* ^3−^02140^+^	Triple insertional knockout leaving one functional GerXA *gerXA4*
	*gerXA2,*	CLOSPO_03006,	795s,		
	*gerXA3*	CLOSPO_02217	61s		
	*gerXA1,*	CLOSPO_00838,	175s,	*gerXA* ^3−^02217^+^	Triple insertional knockout leaving one functional GerXA *gerXA3*
	*gerXA2,*	CLOSPO_03006,	795s,		
	*gerXA4*	CLOSPO_02140	601s		
	*gerXA1,*	CLOSPO_00838,	175s,	*gerXA* ^3−^03006^+^	Triple insertional knockout leaving one functional GerXA *gerXA2*
	*gerXA3,*	CLOSPO_02217,	61s,		
	*gerXA4*	CLOSPO_02140	601s		
	*gerXA2,*	CLOSPO_03006,	795s,	*gerXA* ^3−^00838^+^	Triple insertional knockout leaving one functional GerXA *gerXA1*
	*gerXA3,*	CLOSPO_02217,	61s,		
	*gerXA4*	CLOSPO_02140	601s		
	*gerXA1,*	CLOSPO_00838,	175s,	*gerXA* ^4−^	Quadruple insertional knockout. Insertion in all known *gerXA* sub-units
	*gerXA2,*	CLOSPO_03006,	795s,		
	*gerXA3,*	CLOSPO_02217,	61s,		
	*gerXA4*	CLOSPO_02140	601s		
***Plasmids***	pMTL007C-E2				Clostridial mutagenesis plasmid
	pMTL8315esp				GR expression plasmid with no fdx promoter. Relies on native promoter of inserted genes
	pMTL8315fdx				GR expression plasmid with fdx promoter

− indicates the gene is absent; + indicates that only that gene is functional; ^no−^ describes the number of genes knocked out. a = antisense orientation insertion site; s = sense orientation insertion site. The designation of “*X*” before the letter A is used in clostridia as unlike *Bacillus*, GRs in these species have not yet been attributed to specific germinants.

### Germinants and spore germination

The potential germinants, including; L-alanine, L-serine, L-cysteine, L-methionine, L-phenylalanine, glycine (0.5 mM–100 mM) and antagonists d-alanine, D-cysteine, D-methionine, D-phenylalanine, D-serine (10 mM–200 mM depending on solubility) (Sigma) were all prepared in Tris-HCl buffer (20 mM, pH 7.4) with NaHCO_3_ (50 mM), with or without L-lactate (50 mM). NaHCO_3_ was a non-essential component that increases the rate and overall extent of germination by approximately 10% [54]. Germinant solutions were prepared and filter sterilised (0.45-µm syringe filter, Millipore, Bedford, MA). The pH of the germinant solutions was adjusted to evaluate the effect of pH on spore germination at pH 3 to pH 10. Prior to the addition of germinants all spore suspensions were heat activated at 80°C for 10 min. Germination of spores at 30°C was measured by a decrease in optical density (OD) at 600 nm every 5 min using a Bioscreen C analyser system (Labsystems, Basingstoke, UK) under aerobic conditions. Germination was expressed in terms of measured OD_600_ as a percentage of the initial OD_600_. To validate the OD_600_ measurements, at the completion of each test the proportion of germinated spores was visualised by the assessment of 200 spores in at least ten fields using phase-contrast microscopy. Typically, full germination was indicated when the OD_600_ fell to ∼40% of its initial value. In some tests a small fall in OD_600_ was observed (<10% of initial value). This was attributed to settling of spores in the Bioscreen wells, and was not accompanied by microscopic observation of spore germination. Finally, the capacity of spores to germinate and form colonies was assessed. Spore suspensions were enumerated using a haemocytometer and adjusted to a final concentration of ∼1×10^8^ spores/ml. Spores were then heat activated (80°C, 15 min), serially diluted in 0.85% saline, and plated in triplicate on to TY agar before incubation anaerobically (37°C, 48 hrs).

### Mutant generation

*Clostridium* mutants were generated using the Clostron system, which inserts an erythromycin resistance cassette into the targeted gene of interest. Target sites were identified using the Pertuka method [80] and mutants were generated (Table 2) as described by Heap *et al.* [81]. *gerX*A GR subunits were targeted in all germination operons. Re-targeted introns were ligated into the pMTL007C-E2 vector following restriction digest with *Hin*dIII/*Bsr*GI. All retargeted introns were sequence verified before transformation into *E. coli* CA434. Confirmed sequenced plasmids were then conjugated into their respective hosts. Finally, primers were designed and used to confirm that the intron was present and in the correct orientation in the target gene/genes of interest (Table S2). The current insertional knockout system does not allow selective isolation of clones containing a second intron insertion as following one insertion the mutant strain is then erythromycin resistant. To create the double, triple and quadruple insertional knockout mutants an alternative approach was taken in which clones containing intron insertions were identified by screening large numbers of colonies rather than by antibiotic selection. Plasmid re-targeting was carried out as above and transferred in to *C. botulinum* or *C. sporogenes* using *E. coli* CA434. Confirmation of successful trans-conjugation events were screened on TY agar plates containing cycloserine (250 µg/ml) and thiamphenicol (15 µg/ml). To create the multiple insertional knockout mutants the process was repeated using successive rounds of plasmid targeting and gene insertion. Successful integration of introns into the target genes was confirmed by PCR using primers flanking the target sites (Table S2). Furthermore, to confirm that the required number of insertion events had occurred, genomic DNA from the mutants was digested with *Hin*dIII and subjected to Southern analysis using an intron specific probe.

### Complementation

For complementation, two plasmids, pMTL8315esp and pMTL8315fdx were constructed (Figure S2). Primers, with *Esp*3I restriction sites were designed to amplify two GR fragments; one included the 5′ noncoding region encompassing the putative promoter and was ligated into pMTL8315esp; the second fragment contained the coding operon only and was ligated into the plasmid pMTL8315-fdx which contains the strong promoter P*_fdx_* of the ferredoxin gene (*fdx*) from *C. sporogenes* NCIMB 10696. Following confirmation by sequencing, GR plasmids were then transconjugated into their respective mutants using *E. coli* CA434 as described earlier. Spore crops were produced as above, except with the addition of thiamphenicol (15 µg/ml) to the media to maintain the plasmid.

## Supporting Information

Figure S1 **Confirmation of successful insertional mutagenesis.** **A**, PCR screens of *C. botulinum* ger gene mutants confirms that the intron has successfully inserted into the target gene. Primer set 1 anneals to the ErmRAM and confirms that the RAM is spliced and therefore integrated (expected band size of mutants 900 bp). Primer set 2, intron binding EBS universal primer plus gene specific forward primer amplified sequence across the intron-exon junction to confirm intron insertion into the desired gene (expected band size of mutants 250 bp). Primer set 3 anneals to each end of the target gene and confirms the intron is present in the target gene (expected band size of mutants ∼2 kb). Lanes labelled WT show the PCR products (primer set 3) generated for each ger A gene when wild type DNA is used as template. **B**, Diagrammatic representation of results shown in **A** revealing primer binding sites and length of pcr products. **C**, Southern hybridisation was performed to confirm the correct number of insertion events had occurred. The hybridisation probe was designed to target the chromosomally inserted ErmRAM sequence. Genomic DNA of all strains was digested overnight with *HindIII*. Expected band sizes were as follows; gerXA1-0123^−^ 2.9 kb, gerXA2-1975^−^ 6 kb, gerXA3-2797^−^ 4 kb. The negative control, WT genomic DNA, generated no signal for the probe.
(PDF)

Figure S2 **Constructed plasmids used for expression of** ***Clostridium*** **receptors in complementation experiments.** Complementation plasmid components; catP (chloramphenicol acetyltransferase) conferring thiamphenicol resistance in *E.coli* and chloramphenicol resistance in *Clostridium*. repH (pCB102); Gram-positive replicon. ColE1; Gram-negative replicon. traJ; plasmid conjugative transfer protein. *Esp3I*; restriction enzyme site used for insertion of GR operons. Plasmid pMTL8315fdx contains the strong promoter P_fdx_ of the ferredoxin gene (fdx) from *C. sporogenes* NCIMB 10696. Plasmid pMTL8315esp contains no additional promoter and relies on a wild type copy of the inserted gene under the control of its native promoter (assumed to be located in the intergenic region upstream of the first CDS of the operon). Both plasmids are adaptations of the pMTL80000 modular plasmid system of Heap et al., 2009 [82].
(PDF)

Table S1 **Effect of amino acid concentrations on clostridial spore germination.** * *C. botulinum* only, no germination detected in *C. sporogenes* at 100 mM. A selection of amino acids, were assessed for their individual effect on germination of spores of *C. botulinum* and *C. sporogenes* in the presence of Tris-HCl buffer (pH 7.4), L-lactate (50 mM) and NaHCO_3_ (50 mM). Germination was determined by a decrease in optical density (OD) at 600 nm using a Bioscreen C analyser and validated by phase-contrast microscopy.
(PDF)

Table S2 **Primers used in this study.** + denotes resulting product contains >500 bp upstream of the named gene to be certain that a native promoter is included.
(PDF)
