# Supplementary material for: Functional Characterisation of Germinant Receptors in Clostridium botulinum and Clostridium sporogenes Presents Novel Insights into Spore Germination Systems
Source: PLoS Pathog. 2014 Sep 11;10(9):e1004382. doi: 10.1371/journal.ppat.1004382 (PMC4161481; doi:10.1371/journal.ppat.1004382)
Supplement: Figure S1 — Confirmation of successful insertional mutagenesis. A, PCR screens of C. botulinum ger gene mutants confirms that the intron has successfully inserted into the target gene. Primer set 1 anneals to the ErmRAM and confirms that the RAM is spliced and therefore integrated (expected band size of mutants 900 bp). Primer set 2, intron binding EBS universal primer plus gene specific forward primer amplified sequence across the intron-exon junction to confirm intron insertion into the desired gene (expected band size of mutants 250 bp). Primer set 3 anneals to each end of the target gene and confirms the intron is present in the target gene (expected band size of mutants ∼2 kb). Lanes labelled WT show the PCR products (primer set 3) generated for each ger A gene when wild type DNA is used as template. B, Diagrammatic representation of results shown in A revealing primer binding sites and length of pcr products. C, Southern hybridisation was performed to confirm the correct number of insertion events had occurred. The hybridisation probe was designed to target the chromosomally inserted ErmRAM sequence. Genomic DNA of all strains was digested overnight with HindIII. Expected band sizes were as follows; gerXA1-0123− 2.9 kb, gerXA2-1975− 6 kb, gerXA3-2797− 4 kb. The negative control, WT genomic DNA, generated no signal for the probe. (PDF) [file ppat.1004382.s001.pdf]

Figure S1. Confirmation of successful insertional mutagenesis.

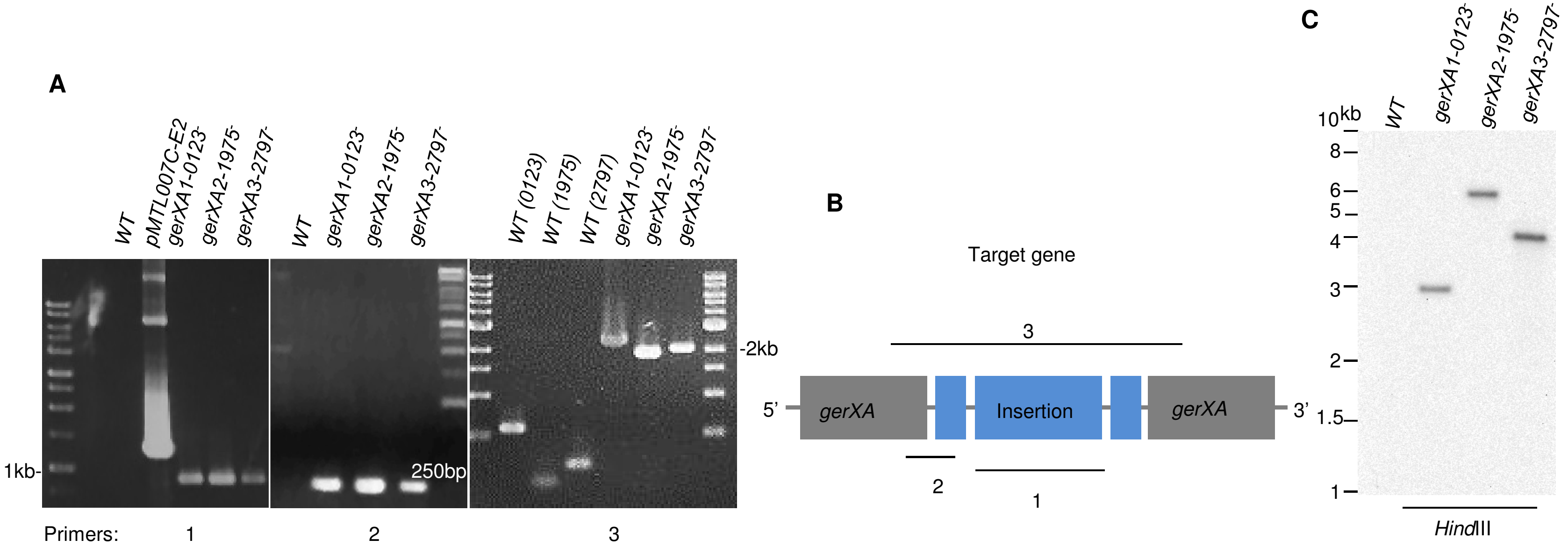

A, PCR screens of *C. botulinum ger* gene mutants confirms that the intron has successfully inserted into the target gene. Primer set 1 anneals to the ErmRAM and confirms that the RAM is spliced and therefore integrated (expected band size of mutants 900bp). Primer set 2, Intron binding EBS universal primer plus gene specific forward primer amplified sequence across the intron-exon junction to confirm intron insertion into the desired gene (expected band size of mutants 250bp). Primer set 3 anneals to each end of the target gene and confirms the intron are present in the target gene (expected band size of mutants ~ 2kb). Lanes labelled WT show the PCR products (primer set 3) generated for each ger A gene when wild type DNA is used as template. **B**, Diagrammatic representation of results shown in **A** revealing primer binding sites and length of pcr products. **C**, Southern hybridisation was performed to confirm the correct number of insertion events had occurred. The hybridisation probe was designed to target the chromosomally inserted ErmRAM sequence. Genomic DNA of all strains was digested overnight with *Hin*dIII. Expected band sizes were as follows; gerXA1-0123$^-$ 2.9kb, gerXA2-1975$^-$ 6kb, gerXA3-2797$^-$ 4kb. The negative control, WT genomic DNA, generated no signal for the probe.
