# Supplementary material for: Functional Characterisation of Germinant Receptors in Clostridium botulinum and Clostridium sporogenes Presents Novel Insights into Spore Germination Systems
Source: PLoS Pathog. 2014 Sep 11;10(9):e1004382. doi: 10.1371/journal.ppat.1004382 (PMC4161481; doi:10.1371/journal.ppat.1004382)
Supplement: Figure S2 — Constructed plasmids used for expression of Clostridium receptors in complementation experiments. Complementation plasmid components; catP (chloramphenicol acetyltransferase) conferring thiamphenicol resistance in E.coli and chloramphenicol resistance in Clostridium. repH (pCB102); Gram-positive replicon. ColE1; Gram-negative replicon. traJ; plasmid conjugative transfer protein. Esp3I; restriction enzyme site used for insertion of GR operons. Plasmid pMTL8315fdx contains the strong promoter Pfdx of the ferredoxin gene (fdx) from C. sporogenes NCIMB 10696. Plasmid pMTL8315esp contains no additional promoter and relies on a wild type copy of the inserted gene under the control of its native promoter (assumed to be located in the intergenic region upstream of the first CDS of the operon). Both plasmids are adaptations of the pMTL80000 modular plasmid system of Heap et al., 2009 [82]. (PDF) [file ppat.1004382.s002.pdf]

Figure S2. Constructed plasmids used for expression of *Clostridium* receptors in complementation experiments.

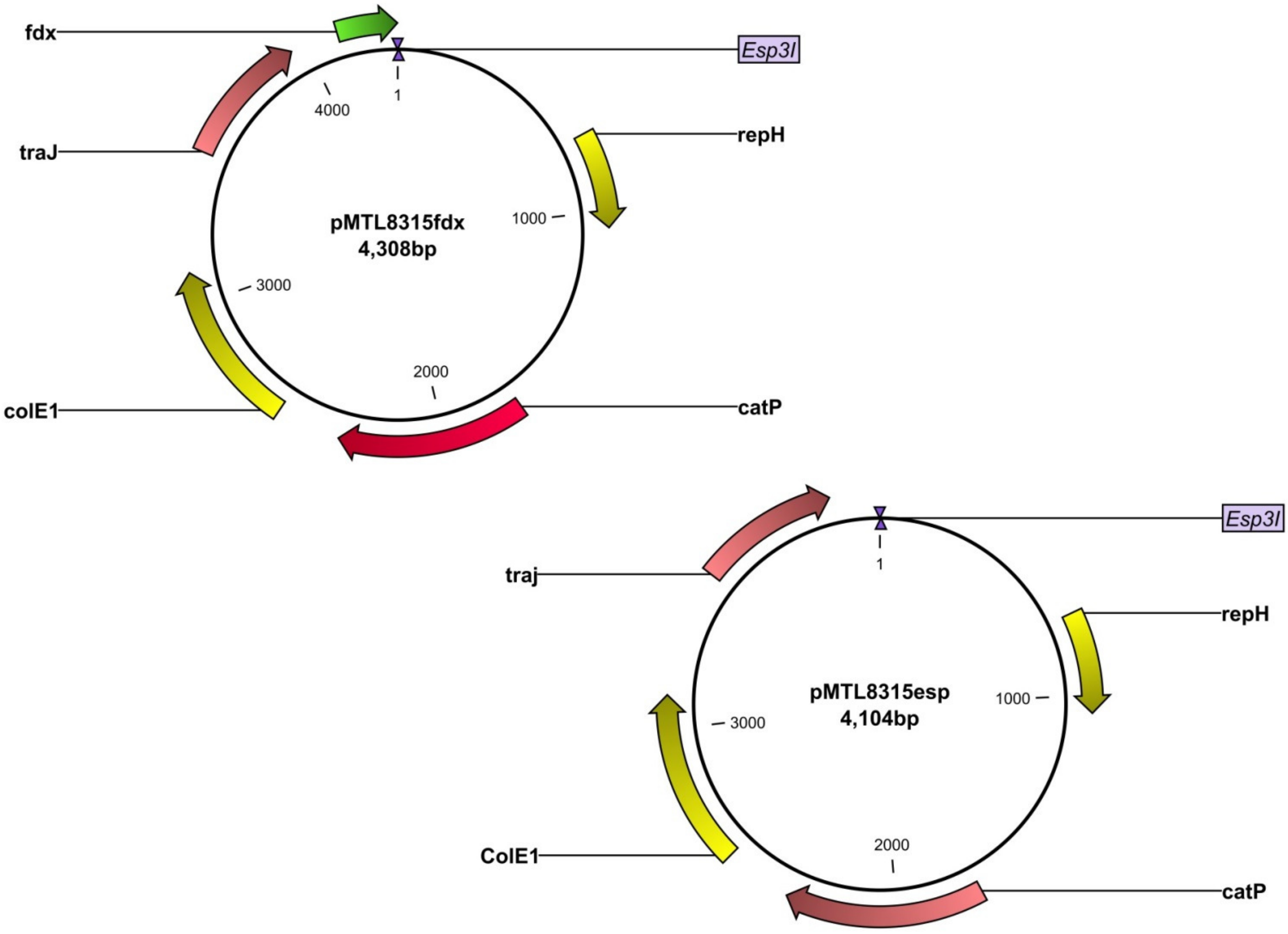

Complementation plasmid components; catP (chloramphenicol acetyltransferase) conferring thiamphenicol resistance in *E.coli* and chloramphenicol resistance in *Clostridium*. repH (pCB102); Gram-positive replicon. ColE1; Gram-negative replicon. traJ; plasmid conjugative transfer protein. *Esp*3I; restriction enzyme site used for insertion of GR operons. Plasmid pMTL8315fdx contains the strong promoter P_{fdx} of the ferredoxin gene (*fdx*) from *C. sporogenes* NCIMB 10696. Plasmid pMTL8315esp contains no additional promoter and relies on a wild type copy of the inserted gene under the control of its native promoter (assumed to be located in the intergenic region upstream of the first CDS of the operon). Both plasmids are adaptations of the pMTL80000 modular plasmid system of Heap et al., 2009.

Heap JT, Pennington OJ, Cartman ST, Minton NP (2009) A modular system for Clostridium shuttle plasmids. J Microbiol Methods 78: 79-85.
