# Supplementary material for: Functional Characterisation of Germinant Receptors in Clostridium botulinum and Clostridium sporogenes Presents Novel Insights into Spore Germination Systems
Source: PLoS Pathog. 2014 Sep 11;10(9):e1004382. doi: 10.1371/journal.ppat.1004382 (PMC4161481; doi:10.1371/journal.ppat.1004382)
Supplement: Table S1 — Effect of amino acid concentrations on clostridial spore germination. * C. botulinum only, no germination detected in C. sporogenes at 100 mM. A selection of amino acids, were assessed for their individual effect on germination of spores of C. botulinum and C. sporogenes in the presence of Tris-HCl buffer (pH 7.4), L-lactate (50 mM) and NaHCO3 (50 mM). Germination was determined by a decrease in optical density (OD) at 600 nm using a Bioscreen C analyser and validated by phase-contrast microscopy. (PDF) [file ppat.1004382.s003.pdf]

Table S1. Effect of amino acid concentrations on clostridial spore germination.

Potential Germinants	Germination	Minimum concentration tested at which germination occurs
L-alanine	+ve	0.5mM
L-cysteine	+ve	10mM
L-phenylalanine	+ve	10mM
L-serine	+ve	10mM
L-methionine	+ve	10mM
Glycine	+ve*	20mM
Inosine	-ve	No germination at 100mM
AGFK (L-asparagine, glucose, fructose, and potassium ions)	-ve	No germination at 100mM
L-asparagine	-ve	No germination at 100mM
Nicotinamide	-ve	No germination at 100mM
L-proline	-ve	No germination at 100mM
L-leucine	-ve	No germination at 100mM
L-isoleucine	-ve	No germination at 100mM
L-arginine	-ve	No germination at 100mM
L-glutamine	-ve	No germination at 100mM
L-aspartic acid	-ve	No germination at 100mM
L-lysine	-ve	No germination at 100mM
L-valine	-ve	No germination at 100mM

* *C. botulinum* only, no germination detected in *C. sporogenes* at 100mM.
A selection of amino acids, were assessed for their individual effect on germination of spores of *C. botulinum* and *C. sporogenes* in the presence of Tris-HCl buffer (pH 7.4), L-lactate (50mM) and $NaHCO_3$ (50mM). Germination was determined by a decrease in optical density (OD) at 600nm using a Bioscreen C analyser and validated by phase-contrast microscopy.
