# Supplementary material for: Functional Characterisation of Germinant Receptors in Clostridium botulinum and Clostridium sporogenes Presents Novel Insights into Spore Germination Systems
Source: PLoS Pathog. 2014 Sep 11;10(9):e1004382. doi: 10.1371/journal.ppat.1004382 (PMC4161481; doi:10.1371/journal.ppat.1004382)
Supplement: Table S2 — Primers used in this study. + denotes resulting product contains >500 bp upstream of the named gene to be certain that a native promoter is included. (PDF) [file ppat.1004382.s004.pdf]

Table S2. Primers used in this study

Primers/function	Sequence
Southern Blot probe	
Erm-F	5'-CCGATACCGTTTACGAAATTGGAACAGG-3'
Erm-R	5'-TTATTTCCTCCCGTTAAATAATAGATAACT-3'
Insert confirmation	
CBO0123-F	5'-GTTAATCGAGGGGGGTATTACTATG-3'
CBO0123-R	5'-GCTGAAGCTACTACGTCTGG-3'
CBO1975-F	5'-GCCTGTTATCACCATAACTCAAAG-3'
CBO1975-R	5'-GCGCCTATGAACTTAAAGGG-3'
CBO2797-F	5'-CCTCATCTCATTCACTATCTTCTC-3'
CBO2797-R	5'-GGCACAGGAAAGTATTGGCTAAA-3'
CLOSPO_03006-F	5'-GGGTTAACTAATAACCAAGTCATAGATAGAG-3'
CLOSPO_03006-R	5'-AAAAAACCATCCTATGGTAATGCCGAACATGCCTAA-3'
CLOSPO_02140-F	5'-GTATTTAGAGATGTTTCTTGTAAACAAGGAACCCTAC-3'
CLOSPO_02140-R	5'-TATAACCATGGTAGATTGCGATGTTAAGCT-3'
CLOSPO_02217-F	5'-ATTATGCTAGACAGGGGATTTATACAAAATGGATACG-3'
CLOSPO_02217-R	5'-CTATCTCCATTACTGGACTGAAAGCAATCC-3'
EBS universal	5'-CGAAATTAGAAACTTGCGTTCAGTAAAC-3'
ErmRAM-F	5'-TATATTGATAAAAATAATAATAGTGGG-3'
ErmRAM-R	5'-GCGACTCATAGAATTATTTCCTCCCG-3'
Receptor construction	
CLOSPO_02217-F	5'-ATCGTCTCACATGAGAGATAAATTAGATGA-3'
CLOSPO_02219-R	5'-ATCGTCTCACATGTTAGAAAGTTCCTCTAACCC-3'
CLOSPO_02217-F+	5'-ATCGTCTCACATGTAATTCTAGATATGGTAGAATGC-3'
CLOSPO_00836-R	5'-ATCGTCTCACATGTTATTTTATTGCTCCTATTC-3'
CLOSPO_00838-F	5'-ATCGTCTCACATGAAGGAAATAGTTAAGGA-3'
CLOSPO_00839-F+	5'-ATCGTCTCACATGGGAAGAATAATAGGTAT-3'
CLOSPO_03006-F	5'-ATCGTCTCACATGGTGTTTATGAGAAATAG-3'
CLOSPO_03003-R	5'-ATCGTCTCACATGTTATGAATTAGGTTGGAGTA-3'
CLOSPO_03006-F+	5'-ATCGTCTCACATGTTATTTTCATAAATTTACTA-3'
CLOSPO_02140-F	5'-ATCGTCTCACATGTTAATATCTTCTAGCCTTATATGG-3'
CLOSPO_02139-R	5'-ATCGTCTCACATGTTATGAAATAATATCAGCAC-3'
CBO0123-F	5'-ATCGTCTCACATGGTGTATAAGATGAAGGAAGTAG-3'
CBO0125-R	5'-ATCGTCTCACATGTTATTTTATTCCTCCTATTC-3'
CBO0123-F+	5'-ATCGTCTCACATGGGAAGAATAATAGGCATATTTG-3'
CBO1974-F	5'-ATCGTCTCACATGTTGAAAAAAATTTGTTTAGG-3'
CBO1978-R	5'-ATCGTCTCACATGTTATGAATTAGATTGGAGTA-3'
CBO1974-F+	5'-ATCGTCTCACATGTATTTTAATAAACTTACTACATTATAC-3'
CBO02797-R	5'-ATCGTCTCACATGTTAAAAGGTTCCTCTGCCACCAC-3'
CBO02795-F	5'-ATCGTCTCACATGAGAGATAAGTTAGATAA-3'
CBO02798-F+	5'-ATCGTCTCACATGGTGTGCGCTATGCTAGACAGAGG-3'
Plasmid primers	
Sbf-F	5'-GCGCGTGATTGCCAAGCA-3'
Asc-R	5'-TCTGTAAATTTCTTTCTATTCAGCACTGT-3'

+ denotes resulting product contains >500bp upstream of the named gene to be certain that a native promoter is included.
